# Effects of morbidity on smoking cessation: a national smoking cessation program

**DOI:** 10.1186/s13722-021-00257-3

**Published:** 2021-07-28

**Authors:** Young-Gyun Seo, Min-Woo Jo, Yu-Jin Paek, Jaekyung Choi

**Affiliations:** 1 Department of Family Medicine, Hallym University Sacred Heart Hospital, Anyang, Republic of Korea; 2 Department of Preventive Medicine, University of Ulsan College of Medicine, 88, Olympic-ro 43-gil, Songpa-gu, Seoul, 05505 Republic of Korea; 3 Department of Family Medicine, Research Institute of Medical Science, School of Medicine, Konkuk University, Konkuk University Medical Center, Seoul, Republic of Korea

**Keywords:** Smoking cessation, Chronic disease, Hypertension, Diabetes mellitus, Pulmonary disease, Depressive disorder

## Abstract

**Background:**

In smokers with chronic diseases, we examined the abstinence rates over 6 months and its affecting factors in the context of the Korea National Health Insurance Service (NHIS) smoking cessation program.

**Methods:**

To identify 6-month abstinence, we extracted a sample of 15,017 participants using the multi-stage stratified cluster sampling method from the 359,047 individuals enrolled in the 2016 NHIS smoking cessation program and 1500 smokers responded to the telephone survey. From this group, 1245 individuals (48.50 ± 12.55 years; men 89.8%) were enrolled, as they had no missing information for confounding variables. We compared chronic disease distribution between participants and current smokers in the 2016 Korea National Health and Nutrition Examination Survey. We evaluated the factors affecting continuous abstinence rate (CAR) across patients with different chronic diseases: hypertension, diabetes mellitus (DM), dyslipidemia (DL), chronic obstructive pulmonary disease, and major depressive disorder (MDD).

**Results:**

While participation of DM patients was high, the participation of DL patients was relatively low. The CAR over 6 months was 44.74%. The adjusted odds ratio (OR) for continuous abstinence over 6 months was significantly lower in the MDD group than in the no-MDD group (OR 0.43, 95% confidence interval [CI] 0.21 to 0.85). The factors of program completion (complete versus incomplete: OR 3.11, 95% CI 2.43 to 3.98), region (non-metropolitan areas versus Seoul metropolitan area: OR 1.28, 95% CI 1.01 to 1.61), and nicotine dependence (severe versus light or moderate: OR 0.64, 95% CI 0.50 to 0.83) were significantly associated with CAR.

**Conclusions:**

The smoking cessation program was not actively recruiting smokers with chronic diseases. The CARs in each disease group were not different from those in the non-disease groups, except that the MDD group had a lower CAR over 6 months than the no-MDD group. Recruiting smokers with chronic diseases and improving their CARs depends on the careful identification of their characteristics.

## Background

It is well known that smoking has negative health impacts and results in increased mortality. It has been reported to cause or increase the risk of cardiovascular and respiratory diseases, various types of cancer, and diabetes mellitus (DM) [1–4]. Moreover, recent data has shown that a total of 52 diseases are associated with smoking [5]. Globally, 6 million people die each year from smoking, with second-hand smoke causing an additional 6 million deaths [6]. In South Korea (hereafter Korea), the number of deaths related to smoking was estimated at 58,155 of about 260,000 yearly deaths among those aged 30 years and older [7].

Therefore, reducing the smoking rate is an important healthcare task in most countries. In January 2015, as part of its pricing policy, the Korean government raised the price of tobacco [8]. The following month, it launched the ‘National Health Insurance Services (NHIS) smoking cessation program’ (a non-pricing policy) to improve the national rate of smoking cessation and support smokers’ efforts to quit [9]. All Korean citizens can join the NHIS smoking cessation program. This program provides financial support for smokers to obtain physician consultations and purchase smoking cessation medications, including nicotine-based supplements. In the 1st year of the program, the self-reported 6-month continuous abstinence rate (CAR) was 30.5% [10].

Prior to 2015, only about 250 public health centers in local areas of Korea operated smoking cessation clinics. The NHIS smoking cessation program differs from the previously established programs in enabling participating general clinics and hospitals to prescribe smoking cessation medications, such as varenicline and bupropion [9]. In 2016, 10,468 organizations participated in the program, greatly improving its overall accessibility. As a result, the program could expand its focus from smokers visiting a specific medical facility for the purpose of smoking cessation to those visiting facilities for any other purpose. Smokers could learn about the opportunity to enroll in the program during one of these visits. Particularly, the expansion was important to induce more individuals across the nation to participate in smoking cessation treatment.

Smoking amplifies the risk of cardiovascular events in patients with chronic conditions, such as DM and hypertensive diseases (HTN). This is true even if these patients’ relative risk for cardiovascular events is not significantly higher than those of individuals without chronic diseases [11]. Improving accessibility to the programs is critical. It allows for the recruitment of patients who visit clinics for reasons other than smoking cessation and whose motivation to stop smoking may be relatively low compared to that of voluntary program participants. In this context, analyzing the characteristics of smoking cessation in chronically ill smokers, including those who visit clinics regularly as well as those who visit irregularly, is needed. However, there are few studies on the effects of chronic diseases on smoking cessation or the differences in abstinence rate across patients with different chronic diseases. Thus, we examined the effects of the NHIS smoking cessation program on and the factors influencing smoker abstinence rates across patients with several chronic diseases.

## Methods

### Participants

**Fig. 1:**
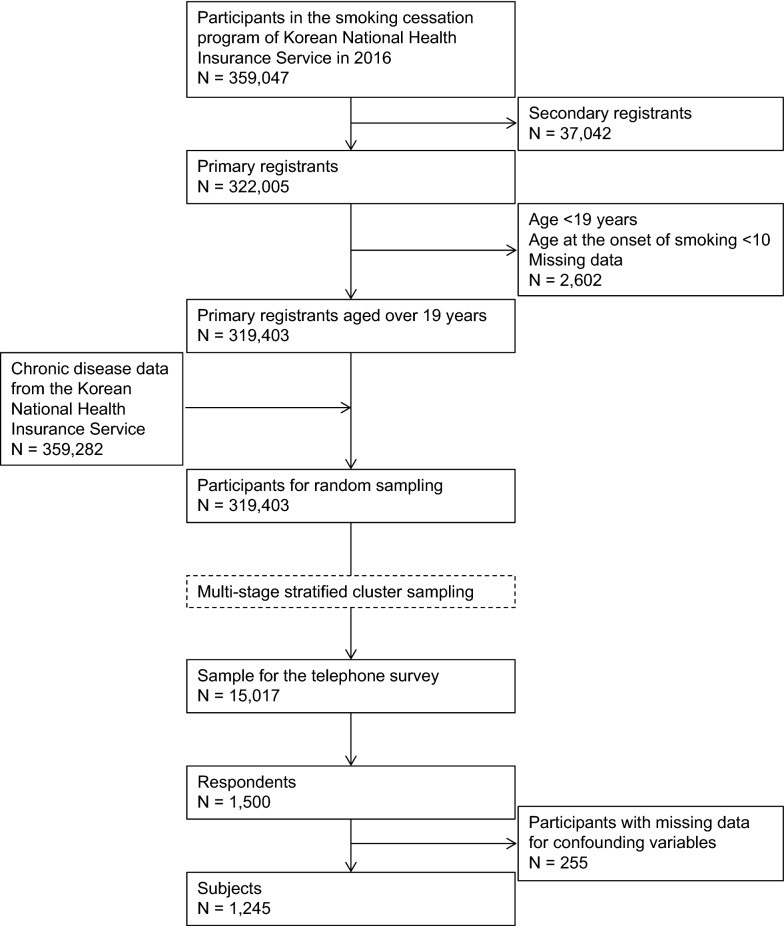
Selection process of the participants. In total, 359,047 people were enrolled in the Health Insurance Service’s smoking cessation program in 2016. A total of 1245 participants (1118 men, 89.8%) were selected for analysis using a multi-stage stratified cluster sampling method

In total, 359,047 people were enrolled in the 2016 NHIS smoking cessation program. From this group, we excluded second registrants, those under 19 years of age, those who started smoking before the age of 10, and those with data errors, resulting in the inclusion of 319,403 individuals. These individuals’ data were linked with chronic disease claims data from the NHIS. Then, based on stepwise population statistics (the number of cases and the composition ratio), we used random sampling to extract a sample for telephone survey. We calculated the proportion of individuals who completed the smoking cessation program in 64 layers by sex (2), age (4), and region variables (8), and we performed oversampling so that program completion was doubled in each layer. Based on an expected response rate of 10%, we extracted a sample of 15,017 participants (10 times the target sample size of 1500) using the multi-stage stratified cluster sampling method as described above. Of this group, 1500 smokers responded to the telephone survey, but we excluded participants who did not provide data on the confounding variables of income level, hospital type, nicotine dependence level, duration of smoking, and type of smoking cessation medication. In the end, a total of 1245 participants (1118 men, 89.8%) were considered in the analysis (Fig. 1).

This study was conducted according to the guidelines established by the Declaration of Helsinki. The study protocol was approved by the Institutional Review Board of Hallym University Sacred Heart Hospital (Approval number: 2018-07-007). The need for informed consent was waived on account of the retrospective nature of the National Health Information Database.

### National Health Insurance Service smoking cessation program

The NHIS smoking cessation program provided financial support for physician consultation fees (up to six visits) and for purchasing smoking cessation medications (varenicline or bupropion) or smoking cessation supplements (nicotine patch, gum, or lozenges) for 8–12 weeks after registration on February 25, 2015. A total of two registrations were allowed per year. Program completion was defined as either the completion of six physician consultations or 8–12 weeks of medication.

### Diagnosis codes

HTN, DM, and disorders of lipoprotein metabolism and other lipidemias (DL) were defined by the following ICD-10-CM codes, respectively: I10–I15, E10–E14, and E78. Cardiovascular disease (CVD) was defined as having been diagnosed with one or more among NTH, DM, and DL. The number of CVD was defined as the number of diagnosed diseases among NTH, DM, and DL. Chronic obstructive pulmonary disease (COPD) and major depressive disorder (MDD) were defined by the following ICD-10-CM codes, respectively: J42–J46 and F32 and F33. We extracted data on diseases from January 1, 2014 to December 31, 2015. We defined a participant with the condition as having been diagnosed at least once prior to the survey.

### Income level

Korea has a national health insurance system, and its insurer is the NHIS. Because the NHIS imposes premiums according to income levels, we used premium payment as a surrogate marker for income level. We defined the low-income (LI) group to include participants with premium payments in the lowest 20% and the middle or high-income (MHI) group to include participants with premium payments in the upper 80%.

### Other influencing factors

Baseline data extracted from the National Health Information Database of the NHIS in 2016 included data on the following: region (Seoul metropolitan region or others); health facility type (community clinics or others); program completion status; nicotine dependence level according to the Fagerstrom Test for Nicotine Dependence (FTND) score with a score of 0–3 indicating light dependence, 4–6 as moderate dependence, and 7–10 as severe dependence; duration of smoking; and the type of smoking cessation medication (varenicline, bupropion, and nicotine replacement therapy).

### Telephone survey for assessing smoking cessation

A telephone survey was conducted at least 6 months after the first smoking cessation consultation with a physician. The abstinence rates were derived from participants’ responses to the question: ‘How long did you maintain smoking cessation status after registering for the NHIS smoking cessation program in 2016?’ The responses were less than 1 month, more than 1 month, more than 3 months, or more than 6 months. We defined ‘less than 1 month’, ‘more than 1 month’, or ‘more than 3 months’ as ‘continuous abstinence of less than 6 months’, and we defined ‘more than 6 months’ as ‘continuous abstinence over 6 months’.

### The Korea National Health and Nutrition Examination Survey

The Korea National Health and Nutrition Examination Survey (KNHANES) is conducted by the government and is a national survey to evaluate the health and nutritional status of the Korean people [12]. Currently, the KNHANES reports annual health indicators such as smoking rates, disease prevalence, and the health-related quality of life of the general Korean population. We compared several characteristics of our participants with those of current smokers from the 2016 KNHANES.

### Statistical analyses

We used the χ^2^ test and the Fisher’s exact test for categorical variables and the t-test for continuous variables to compare participants and total respondents or participants and KNHANES smokers, respectively. The χ^2^ was also used to compare the CAR over 6 months according to disease types. We performed univariable logistic regression analyses of continuous abstinence over 6 months. Then, multivariable logistic regression analyses were performed after controlling for variables that were identified by univariable analyses or that were clinically significant. In multivariable analyses, adjusting factors were age and sex or age, sex, income level, region, hospital type, program completion status, nicotine dependence level, duration of smoking, type of smoking cessation medication, and chronic diseases (HTN, DM, DL, COPD, and MDD) or age, sex, income level, region, hospital type, program completion status, nicotine dependence level, duration of smoking, type of smoking cessation medication, and chronic diseases (CVD, COPD, and MDD), respectively. In each disease group, we also performed logistic regression analyses after adjusting for age, sex, income level, region, hospital type, program completion status, nicotine dependence level, duration of smoking, and type of smoking cessation medication. We calculated the odds ratios (OR) and 95% confidence intervals (CI) from the logistic regression analyses. All statistical analyses were conducted using Stata/MP, version 14.0 (StataCorp, College Station, TX, USA). All statistical tests were two-sided, and statistical significance was determined at a p-value < 0.05.

## Results

Table 1 presents the characteristics of participants. Characteristics including sex and age of participants were compared with those of the total respondents. The mean age of the participants was 48.50 ± 12.55 years. There were no statistically significant differences in the distributions of age and sex between the two groups.

**Table 1:** Characteristics of participants and total respondents

	Participants (N = 1245)	Total respondents (N = 1500)	p-value^a^
Sex			0.342
Male	1118 (89.80)	1330 (88.67)	
Female	127 (10.20)	170 (11.33)	
Age in years			
Mean ± SD	48.50 ± 12.55	48.90 ± 12.58	0.416
< 50	647 (51.97)	762 (50.80)	0.542
≥ 50	598 (48.03)	738 (49.20)	
Income level			0.921
MHI group	1084 (87.07)	1238 (86.94)	
LI group	161 (12.93)	186 (13.06)	
Region			0.689
Seoul and metropolitan areas	642 (51.57)	762 (50.80)	
Non-metropolitan areas	603 (48.43)	738 (49.20)	
Health care facility type			0.693
Community clinics	946 (75.98)	1130 (75.33)	
Others	299 (24.02)	370 (24.67)	
Program completion status			0.371
Incomplete	767 (61.61)	949 (63.27)	
Complete	478 (38.39)	551 (36.73)	
Nicotine dependence level^b,c^			0.974
Light/moderate	832 (66.83)	907 (66.89)	
Severe	413 (33.17)	449 (33.11)	
Duration of smoking, years^c^	25.91 ± 11.81	26.10 ± 11.84	0.691
Smoking cessation medication^c^			0.941
Varenicline only	1101 (88.43)	1307 (87.90)	
Bupropion only	96 (7.71)	118 (7.94)	
NRT only	13 (1.04)	19 (1.28)	
Others	35 (2.81)	43 (2.89)	
Duration of smoking cessation (months)			0.504
< 6	688 (55.26)	848 (56.53)	
≥ 6	557 (44.74)	652 (43.47)	
Disease			
HTN	241 (19.36)	283 (18.87)	0.745
DM	154 (12.37)	170 (11.33)	0.402
DL	95 (7.63)	109 (7.27)	0.717
No. of CVD			0.875
0	830 (66.67)	1021 (68.07)	
1	345 (27.71)	401 (26.73)	
2	65 (5.22)	73 (4.87)	
3	5 (0.40)	5 (0.33)	
COPD	64 (5.14)	71 (4.73)	0.623
MDD	45 (3.61)	49 (3.27)	0.618

*SD* standard deviation, *MHI* middle or high-income, *LI* low-income, *NRT* nicotine replacement therapy, *HTN* hypertensive diseases, *DM* diabetes mellitus, *DL* disorders of lipoprotein metabolism and other lipidemias, *CVD* cardiovascular diseases (hypertensive diseases, diabetes mellitus, or disorders of lipoprotein metabolism and other lipidemias), *COPD* chronic obstructive pulmonary disease, *MDD* major depressive disorder

Data are presented as mean ± SD or number (%)

^a^p value from a χ^2^ test for binary variables or t-test for continuous variables, comparing differences between any two groups

^b^Nicotine dependence level defined by the FTND score: 0–3, light; 4–6, moderate; 7–10, severe

^c^Different total number due to missing data in total respondents: 1424 for income level; 1356 for nicotine dependence level; 1317 for duration of smoking; 1487 for smoking cessation medication

Table 2 shows the comparison of participants’ characteristics with those of current smokers in the 1st year of KNHANES VII (2016). The proportion of men was higher in participants than in general current smokers (89.80% vs. 83.40%, p < 0.001). The proportion of smokers older than 50 years was relatively low in general current smokers (39.29%) compared to that in participants (48.03%), and the mean age in the general current smoker group was lower than that in the participant group (46.29 years vs. 48.50 years). The distributions of several chronic diseases, except for DM, DL, and CVD, were similar between the two groups. The prevalence of DM was higher in participants than in the general current smokers (12.37% vs. 9.24%, p = 0.015). The prevalence of DL was lower in participants than in the general current smokers (7.63% vs. 12.98%, p < 0.001). The prevalence of CVD was higher in participants than in the general current smokers (33.33% vs. 28.44%, p < 0.001).

**Table 2:** Comparison of participants’ characteristics with those of current smokers in the Korean National Health and Nutrition Examination Survey

	Participants (N = 1245)	Current smokers in the KNHANES (N = 1125)	p-value^a^
Sex			< 0.001
Male	1118 (89.80)	940 (83.56)	
Female	127 (10.20)	185 (16.44)	
Age, year			
Mean ± SD	48.50 ± 12.55	46.29 ± 15.10	< 0.001
< 50	647 (51.97)	683 (60.71)	< 0.001
≥ 50	598 (48.03)	442 (39.29)	
Disease			
HTN	241 (19.36)	232 (20.62)	0.442
DM	154 (12.37)	104 (9.24)	0.015
DL	95 (7.63)	146 (12.98)	< 0.001
No. of CVD			< 0.001
0	830 (66.67)	805 (71.56)	
1	345 (27.71)	188 (16.71)	
2	65 (5.22)	102 (9.07)	
3	5 (0.40)	30 (2.67)	
COPD	64 (5.14)	31 (4.94)	0.849
MDD	45 (3.61)	43 (4.03)	0.600

*KNHANES* Korean National Health and Nutrition Examination Survey, *SD* standard deviation, *HTN* hypertensive diseases, *DM* diabetes mellitus, *DL* disorders of lipoprotein metabolism and other lipidemias, *CVD* cardiovascular diseases (hypertensive diseases, diabetes mellitus, or disorders of lipoprotein metabolism and other lipidemias), *COPD* chronic obstructive pulmonary disease, *MDD* major depressive disorder

^a^p value from a χ^2^ test for binary variables or t-test for continuous variables, comparing differences between any two groups

CARs over 6 months according to disease types are shown in Table 3. CARs over 6 months for patients with HTN, DM, DL, CVD, COPD, and MDD were 48.55%, 45.45%, 46.32%, 46.75%, 45.31%, and 28.89%, respectively. Comparing participants with and without diseases, there was no significant difference in CARs over 6 months except in the case of MDD. Those with MDD had a significantly lower CAR over 6 months than those without MDD (28.89% vs. 45.33%, p = 0.029).

**Table 3:** Continuous abstinence rates over 6 months according to disease type

	HTN		DM		DL		CVD		COPD		MDD	
	No	Yes	No	Yes	No	Yes	No	Yes	No	Yes	No	Yes
Duration of smoking cessation (months)												
< 6	564 (56.18)	124 (51.45)	604 (55.36)	84 (54.55)	637 (55.39)	51 (53.68)	467 (56.27)	221 (53.25)	653 (55.29)	35 (54.69)	656 (54.67)	32 (71.11)
≥ 6	440 (43.82)	117 (48.55)	487 (44.64)	70 (45.45)	513 (44.61)	44 (46.32)	363 (43.73)	194 (46.75)	528 (44.71)	29 (45.31)	544 (45.33)	13 (28.89)
p-value^a^	0.185		0.849		0.748		0.314		0.925		0.029	

Data are presented as number (%)

*HTN* hypertensive diseases, *DM* diabetes mellitus, *DL* disorders of lipoprotein metabolism and other lipidemias, *CVD* cardiovascular diseases (hypertensive diseases, diabetes mellitus, or disorders of lipoprotein metabolism and other lipidemias), *COPD* chronic obstructive pulmonary disease, *MDD* major depressive disorder

^a^p value from a χ^2^ test comparing differences between any two groups

Table 4 presents the logistic regression analyses results for continuous abstinence over 6 months. The odds of continuous abstinence over 6 months were lower in those with MDD than in those without it, even after adjusting for potential confounding factors of age, sex, income level, region, hospital type, program completion status, nicotine dependence level, duration of smoking, type of smoking cessation medication, and chronic diseases (CVD and COPD) (OR 0.43, 95% CI 0.21 to 0.86 in model 3). Multivariable logistic regression analyses (model 3) indicated that the following were significantly associated with continuous abstinence over 6 months: registration in non-metropolitan areas (non-metropolitan areas versus Seoul metropolitan area: OR 1.28, 95% CI 1.01 to 1.61); program completion (complete versus incomplete: OR 3.11, 95% CI 2.43 to 3.98); and light or moderate nicotine dependence (severe versus light or moderate: OR 0.64, 95% CI 0.50 to 0.83).

**Table 4:** Logistic regression analyses of continuous abstinence over 6 months

	Crude	Model 1	Model 2	Model 3
Sex				
Male	1 (reference)	1 (reference)	1 (reference)	1 (reference)
Female	1.04 (0.72 to 1.51)	1.04 (0.72 to 1.50)	1.19 (0.79 to 1.81)	1.19 (0.78 to 1.80)
Age in years				
< 50	1 (reference)	1 (reference)	1 (reference)	1 (reference)
≥ 50	1.31 (1.04 to 1.63)	1.31 (1.04 to 1.63)	1.28 (0.92 to 1.79)	1.27 (0.91 to 1.76)
Income level				
MHI group	1 (reference)	1 (reference)	1 (reference)	1 (reference)
LI group	1.15 (0.83 to 1.61)	1.14 (0.82 to 1.60)	1.22 (0.85 to 1.74)	1.23 (0.86 to 1.75)
Region				
Seoul metropolitan area	1 (reference)	1 (reference)	1 (reference)	1 (reference)
Non-metropolitan areas	1.24 (0.99 to 1.54)	1.23 (0.98 to 1.54)	1.28 (1.01 to 1.62)	1.28 (1.01 to 1.61)
Health care facility type				
Community clinics	1 (reference)	1 (reference)	1 (reference)	1 (reference)
Others	1.29 (0.99 to 1.67)	1.26 (0.97 to 1.64)	1.08 (0.82 to 1.43)	1.09 (0.82 to 1.44)
Program completion status				
Incomplete	1 (reference)	1 (reference)	1 (reference)	1 (reference)
Complete	3.15 (2.49 to 4.00)	3.13 (2.46 to 3.97)	3.09 (2.42 to 3.95)	3.11 (2.43 to 3.98)
Nicotine dependence level^a^				
Light/moderate	1 (reference)	1 (reference)	1 (reference)	1 (reference)
Severe	0.64 (0.50 to 0.82)	0.63 (0.50 to 0.81)	0.64 (0.50 to 0.83)	0.64 (0.50 to 0.83)
Duration of smoking, years	1.01 (1.00 to 1.02)	1.00 (0.99 to 1.02)	1.00 (0.98 to 1.01)	1.00 (0.98 to 1.01)
Smoking cessation medication				
Varenicline only	1 (reference)	1 (reference)	1 (reference)	1 (reference)
Bupropion only	0.62 (0.40 to 0.96)	0.61 (0.39 to 0.94)	0.71 (0.45 to 1.13)	0.72 (0.46 to 1.14)
NRT only	0.53 (0.16 to 1.73)	0.48 (0.15 to 1.59)	0.58 (0.17 to 2.02)	0.57 (0.16 to 1.99)
Others	1.12 (0.57 to 2.20)	1.07 (0.54 to 2.10)	0.88 (0.42 to 1.81)	0.87 (0.42 to 1.80)
HTN				
No	1 (reference)	1 (reference)	1 (reference)	
Yes	1.21 (0.91 to 1.60)	1.12 (0.84 to 1.50)	1.07 (0.79 to 1.46)	
DM				
No	1 (reference)	1 (reference)	1 (reference)	
Yes	1.03 (0.74 to 1.45)	0.93 (0.66 to 1.32)	0.89 (0.62 to 1.29)	
DL				
No	1 (reference)	1 (reference)	1 (reference)	
Yes	1.07 (0.70 to 1.63)	1.01 (0.66 to 1.55)	1.14 (0.73 to 1.78)	
No. of CVD				
0	1 (reference)	1 (reference)		1 (reference)
1	1.11 (0.87 to 1.43)	1.02 (0.78 to 1.33)		1.04 (0.79 to 1.39)
2	1.17 (0.71 to 1.94)	1.04 (0.62 to 1.75)		0.92 (0.53 to 1.60)
3	1.93 (0.32 to 11.61)	1.63 (0.27 to 9.90)		2.17 (0.35 to 13.52)
COPD				
No	1 (reference)	1 (reference)	1 (reference)	1 (reference)
Yes	1.02 (0.62 to 1.70)	0.95 (0.57 to 1.59)	1.02 (0.59 to 1.76)	1.03 (0.60 to 1.77)
MDD				
No	1 (reference)	1 (reference)	1 (reference)	1 (reference)
Yes	0.49 (0.25 to 0.94)	0.47 (0.24 to 0.91)	0.42 (0.21 to 0.84)	0.43 (0.21 to 0.86)

Data are presented as odds ratios (95% confidence interval)

Model 1: adjusted for age and sex

Model 2: model 1 + income level, region, hospital type, program completion status, nicotine dependence level, duration of smoking, type of smoking cessation medication, and chronic diseases (hypertensive diseases, diabetes mellitus, disorders of lipoprotein metabolism and other lipidemias, chronic obstructive pulmonary disease, and major depressive disorder)

Model 3: model 1 + income level, region, hospital type, program completion status, nicotine dependence level, duration of smoking, type of smoking cessation medication, and chronic diseases (number of cardiovascular diseases, chronic obstructive pulmonary disease, and major depressive disorder)

*MHI* middle or high-income, *LI* low-income, *NRT* nicotine replacement therapy, *HTN* hypertensive diseases, *DM* diabetes mellitus, *DL* disorders of lipoprotein metabolism and other lipidemias, *CVD* cardiovascular diseases (hypertensive diseases, diabetes mellitus, or disorders of lipoprotein metabolism and other lipidemias), *COPD* chronic obstructive pulmonary disease, *MDD* major depressive disorder

^a^Nicotine dependence level was defined by the FTND score: 0–3, light; 4–6, moderate; 7–10, severe

Logistic regression analyses of continuous abstinence over 6 months showed some significant factors on smoking cessions according to disease type (Table 5). Program completion was significantly associated with continuous abstinence over 6 months in participants with HTN. Light or moderate nicotine dependence was significantly associated with continuous abstinence over 6 months in participants with DL. Program completion and light or moderate nicotine dependence were significantly associated with continuous abstinence over 6 months in participants with CVD. Light or moderate nicotine dependence and duration of smoking were significantly associated with continuous abstinence over 6 months in participants with COPD.

**Table 5:** Logistic regression analyses of continuous abstinence over 6 months according to disease type

	HTN (N = 241)	DM (N = 152)	DL (N = 94)	CVD (N = 415)	COPD (N = 63)	MDD (N = 43)
Sex						
Male	1 (reference)	1 (reference)	1 (reference)	1 (reference)	1 (reference)	1 (reference)
Female	0.48 (0.18 to 1.30)	2.05 (0.65 to 6.54)	1.28 (0.27 to 6.20)	0.69 (0.34 to 1.42)	0.60 (0.08 to 4.55)	3.16 (0.41 to 24.40)
Age in years						
< 50	1 (reference)	1 (reference)	1 (reference)	1 (reference)	1 (reference)	1 (reference)
≥ 50	1.75 (0.86 to 3.53)	0.81 (0.30 to 2.15)	0.79 (0.22 to 2.83)	1.38 (0.81 to 2.36)	6.13 (0.94 to 40.1)	2.48 (0.30 to 20.29)
Income level						
MHI group	1 (reference)	1 (reference)	1 (reference)	1 (reference)	1 (reference)	1 (reference)
LI group	0.99 (0.46 to 2.14)	0.36 (0.10 to 1.23)	0.90 (0.24 to 3.41)	0.90 (0.50 to 1.63)	1.25 (0.23 to 6.77)	0.74 (0.06 to 8.48)
Region						
Seoul metropolitan area	1 (reference)	1 (reference)	1 (reference)	1 (reference)	1 (reference)	1 (reference)
Non-metropolitan areas	0.92 (0.54 to 1.56)	0.60 (0.30 to 1.18)	1.56 (0.63 to 3.85)	0.88 (0.59 to 1.31)	0.34 (0.09 to 1.21)	2.00 (0.38 to 10.43)
Health care facility type						
Community clinics	1 (reference)	1 (reference)	1 (reference)	1 (reference)	1 (reference)	1 (reference)
Others	1.45 (0.76 to 2.78)	0.54 (0.24 to 1.25)	0.97 (0.36 to 2.62)	1.03 (0.64 to 1.67)	2.61 (0.53 to 12.8)	1.35 (0.22 to 8.20)
Program completion status						
Incomplete	1 (reference)	1 (reference)	1 (reference)	1 (reference)	1 (reference)	1 (reference)
Complete	1.98 (1.15 to 3.42)	1.38 (0.69 to 2.76)	2.14 (0.82 to 5.59)	2.00 (1.32 to 3.03)	3.49 (0.95 to 12.8)	1.81 (0.34 to 9.71)
Nicotine dependence level^a^						
Light/moderate	1 (reference)	1 (reference)	1 (reference)	1 (reference)	1 (reference)	1 (reference)
Severe	0.60 (0.34 to 1.05)	0.71 (0.34 to 1.47)	0.34 (0.14 to 0.83)	0.61 (0.40 to 0.93)	0.22 (0.05 to 0.94)	0.41 (0.08 to 2.29)
Duration of smoking, years	0.99 (0.96 to 1.02)	1.02 (0.98 to 1.06)	1.01 (0.96 to 1.06)	0.99 (0.97 to 1.01)	0.93 (0.87 to 1.00)	1.00 (0.92 to 1.08)
Smoking cessation medication						
Varenicline only	1 (reference)	1 (reference)	1 (reference)	1 (reference)	1 (reference)	1 (reference)
Bupropion only	0.96 (0.38 to 2.38)	0.57 (0.12 to 2.67)	0.44 (0.07 to 2.81)	0.83 (0.39 to 1.75)	1.86 (0.10 to 35.3)	1.53 (0.05 to 43.19)
NRT only	1.46 (0.08 to 26.2)	1	N/A	0.44 (0.04 to 4.38)	N/A	1
Others	1.76 (0.32 to 9.87)	2.66 (0.35 to 19.9)	1	1.16 (0.34 to 3.95)	1	2.79 (0.08 to 101.0)

Data are presented as odds ratios (95% confidence interval)

Adjusted for age, sex, income level, region, hospital type, program completion status, nicotine dependence level, duration of smoking, and type of smoking cessation medication

*HTN* hypertensive diseases, *DM* diabetes mellitus, *DL* disorders of lipoprotein metabolism and other lipidemias, *CVD* cardiovascular diseases (hypertensive diseases, diabetes mellitus, or disorders of lipoprotein metabolism and other lipidemias), *COPD* chronic obstructive pulmonary disease, *MDD* major depressive disorder, *MHI* middle or high-income, *LI* low-income, *NRT* nicotine replacement therapy

^a^Nicotine dependence level was defined by the FTND score: 0–3, light; 4–6, moderate; 7–10, severe

## Discussion

We examined the effects of the 2016 Korea NHIS smoking cessation program on the abstinence rate across patients with different chronic diseases and identified factors influencing abstinence rates. There were no significant differences in disease prevalence between study subject and current smokers from the KNHNES data, except in the cases of DM and DL. While the prevalence of DM was found to be higher in the subjects, DL prevalence was found to be higher in the general current smokers. The CAR over 6 months was found to be significantly lower in smokers with MDD than in those without MDD.

In terms of disease groups, the CARs over 6 months for HTN, DM, DL, CVD, and COPD were not different from those of other participants without a chronic disease. However, the CAR was significantly lower in those with MDD than in those without it. This relation was statistically significant after adjustments for program completion and nicotine dependence level. Smoking is known as a risk factor for depression, but ironically, quitting smoking could also be an aggravating factor for depression [13, 14]. Perhaps, the primary objective of patients with MDD in joining the smoking cessation program was to obtain financial support for depression treatment rather than for smoking cessation itself. Therefore, a careful approach is warranted for smoking cessation treatment in patients with depression, and clinically optimizing MDD treatment before initiating smoking cessation is recommended [15]. In treating smoking cessation, bupropion may be somewhat less effective than combination NRT or varenicline, but it is a reasonable alternative for a patient with MDD. All smokers with psychiatric disorders, including substance use disorders, should be offered tobacco dependence treatment, and clinicians must overcome their reluctance to treat this population [16]. In other chronic disease groups, it showed common influencing factors were similar, but some variables were not statistically significant. In patients with HTN, program completion was an important factor for a smoking cessation. In other disease groups, the coefficients of the program completion in the models were relatively large, although they were not statistically significant. It could be important to encourage participants to continuously maintain their absentee status for a successful smoking cessation. As patients with HTN visit clinics regularly, physicians have more opportunities to meet with them. Nicotine dependence level is also an important factor in patients with DL.

Although the NHIS smoking cessation program was launched successfully, the program was not recruiting participants with chronic diseases actively enough. Total participation increased from 230,800 to 2015 [10] to 359,047 in 2016. In 2017, there were 408,097 participants [9]. However, there were no statistical differences in the prevalence of HTN, COPD, or MDD between the program participants and the general population. Moreover, the prevalence of DL was lower in program participants than in the general population, indicating that perhaps DL patients are not being actively recruited into the smoking cessation program. It could be associated with low advice tendency on smoking cessation by physician in Korea. According to the Korean report of the International Tobacco Control (ITC) policy evaluation project, health professionals advised only 23% of smokers to quit within 6 months [17]. In contrast to the findings for DL, the prevalence of DM was higher in program participants than in the general population. In DM care, smoking cessation is of the utmost importance to facilitate glycemic control and prevent complications [18]. Therefore, physicians who treat DM patients may be more likely to recommend smoking cessation to effectively treat DM itself.

There are three categories of reasons why patients with chronic diseases might not participate in the smoking cessation program: providers, participants, and payment systems. First, in Korea, healthcare providers may be less interested in preventive services such as smoking cessation counselling because most (except for family medicine practitioners) are not trained to provide these services. Instead, specialty training emphasizes the latest medical technology. For example, general surgery training programs focus on surgical skills training [19]. This is not a problem limited to any one specialty; rather, it may be a more general issue, as many specialists have opened their own clinics in Korea [20]. To address this issue, the NHIS and academic societies have developed an educational certificate program in smoking cessation [9]. However, this program would have had a limited effect on physician who provided a smoking cessation treatment. In 2016, while more than 11,000 clinics and hospitals participated in the smoking cessation program, many institutes enrolled fewer than 10 smokers. Secondly, there may be issues regarding the patients themselves. One study reported that Korean adult smokers’ awareness regarding the program was relatively low [21]. While most smokers (88.8%) were aware of a smoking cessation clinic in a public health center, only 36.9% were aware of the NHIS smoking cessation program. In addition, patients with chronic diseases may not be familiar with preventive services; they may only receive prescriptions for medications when visiting clinics. Lastly, there could be system issues. The smoking cessation program’s physician reimbursements may not adequately compensate physicians for taking the extra time to counsel patients, even though the NHIS expects this service. In addition, reimbursements are reduced when physicians write prescriptions for a patient’s chronic disease and smoking cessation in the same visit. A separate software program should be used to process claims related to the smoking cessation program versus claims related to treating a patient’s chronic disease. This could be a barrier against enrolling smokers with chronic diseases into the NHIS smoking cessation program.

In this study, the factors of program completion, region, and nicotine dependence level had statistically significant effects on continuous abstinence over 6 months (model 2 and 3). It is well known that a high level of nicotine dependency is a factor that can impinge on the success of smoking cessation efforts [22], and this study confirmed these results in patients with chronic diseases. Previous Korean and Taiwanese studies have also verified these results [10, 23]. Completion of the program was significantly associated with smoking cessation, consistent with the results of our previous study [10]. This finding may reflect not only patients’ willingness to quit smoking but also the strength of provider efforts, suggesting that providers should monitor participants and encourage them to complete the program. Patients with chronic diseases are accustomed to visiting clinics and hospitals, and in this sense, it may be easier for providers to retain them in the program. Furthermore, in this study, registration in non-metropolitan areas was significantly associated with smoking cessation (although treatment adherence for chronic diseases such as HTN may be higher in urban areas than in the countryside [24]). This finding may due to the rapport between providers and patients in non-metropolitan areas. Sex, age, smoking duration, and type of smoking cessation medication have been known to affect smoking cessation [22], but they were not significant influencing factors in this study. Most participants in the NHIS smoking cessation program were men who took varenicline. This finding may be based on the distribution of the study population and/or a small sample size.

This study had some limitations. First, bias may exist due to gaps in the data from the NHIS smoking cessation program. There were some gaps in the age and income level that have the potential to affect the CAR. Therefore, more accurate data management is needed in NHIS. Second, the CARs were obtained from respondents’ self-reports and thus, may be overestimated, which could nullify the effects of some factors. Third, people who continued cessation may have been more likely to respond to the telephone survey than those who did not, which may have influenced the results. Finally, we used claims data for eliciting patient groups, and this approach may also create a bias.

## Conclusions

The smoking cessation program was not actively recruiting smokers with chronic diseases into the program, although overall participation increased by more than 50% from 2015 to 2016. The CARs in each disease group were not different from those in the non-disease groups, except that participants with MDD had a significantly lower CAR over 6 months than those without MDD. The factors of program completion, region, and nicotine dependence were significantly associated with CAR. Recruiting smokers with chronic diseases and improving their CARs depends on the careful identification of their characteristics.

## Data Availability

The dataset is available from the corresponding author on reasonable request.

## Abbreviations

DM: Diabetes mellitus
NHIS: National Health Insurance Services
CAR: Continuous abstinence rate
HTN: Hypertensive diseases
DL: Dyslipidemia
CVD: Cardiovascular disease
COPD: Chronic obstructive pulmonary disease
MDD: Major depressive disorder
LI: Low-income
MHI: Middle or high-income
FTND: Fagerstrom Test for Nicotine Dependence
KNHANES: Korea National Health and Nutrition Examination Survey
OR: Odds ratios
CI: Confidence intervals
ITC: International Tobacco Control
